# Mixing‐Driven Defects and Composition Evolution in Multi‐Material Metal Additive Manufacturing

**DOI:** 10.1002/advs.75723

**Published:** 2026-05-15

**Authors:** Zhilang Zhang, Steve Gaudez, Mario Togni, Steven Van Petegem, Markus Bambach, Mamzi Afrasiabi

**Affiliations:** ^1^ School of Advanced Manufacturing and Robotics Peking University Beijing China; ^2^ Advanced Manufacturing Lab ETH Zurich Leonhardstr Zurich Switzerland; ^3^ Center for Photon Science Paul Scherrer Institut Forschungsstr Villigen Switzerland

**Keywords:** additive manufacturing, defect formation, high‐fidelity simulation, in situ X‐ray imaging, laser powder bed fusion, multi‐material

## Abstract

Multi‐material laser powder bed fusion (LPBF) enables the fabrication of components with spatially tailored compositions, but reliable process design remains largely limited by an incomplete understanding of melt pool interactions, mixing‐induced instabilities, and defect formation. In this work, we combine operando synchrotron X‐ray radiography with high‐fidelity multi‐material multiphysics modeling to resolve, for the first time, the real‐time coupling between inter‐material mixing, keyhole instability, and pore formation. We show that material mixing fundamentally alters the stability landscape of the keyhole, giving rise to composition‐driven shape transitions and mixing‐induced collapse events that generate large pores. Meanwhile, microscale gas entrapment during forced convection of dissimilar materials produces a distinct class of fine pores. Quantitative mapping of transient composition fields reveals how local alloying gradients govern defect trajectories and melt pool transport. This work establishes a validated mechanistic framework for multi‐material LPBF and provides a quantitative basis for process optimization toward defect‐controlled, compositionally graded metal additive manufacturing.

## Graphical abstract

Operando synchrotron X‐ray imaging coupled with high‐fidelity multiphysics modeling uncovers how inter‐material mixing reshapes keyhole dynamics and drives distinct pore‐formation pathways in multi‐material laser powder bed fusion (LPBF). Composition‐dependent instabilities trigger defects, whereas rescanning suppresses porosity and homogenizes Cu distribution, enabling defect‐controlled and compositionally tailored additive manufacturing.

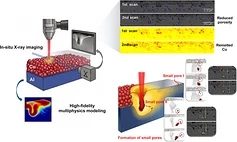

## 1 Introduction

Additive manufacturing (AM) has transformed metal processing by enabling the direct fabrication of geometrically complex components through layer‐wise deposition [1]. Among AM technologies, laser powder bed fusion (LPBF) [2, 3] has emerged as a leading technology for producing high‐precision metallic parts with intricate internal structures. Beyond conventional single‐material builds, recent advances now allow the controlled deposition of multiple alloys within the same component [4]. This capability enables spatial tailoring of thermal, mechanical, or corrosion‐resistance properties, offering new design freedoms for high‐performance applications in aerospace, energy, and biomedical engineering [5, 6, 7, 8]. However, extending LPBF from single‐material to multi‐material processing introduces fundamental scientific challenges arising from the interaction of dissimilar alloys under extreme thermal and fluid dynamic conditions [9, 10].

These challenges arise from the differences in thermophysical, mechanical, and chemical properties of the constituent alloys, which prevent the use of a single optimal set of processing parameters. Thermal expansion mismatch can induce stresses and cracking [11, 12, 13], while segregation and rapid solidification at the interface produce chemical heterogeneities that may lead to the formation of undesired or brittle phases detrimental to interfacial bonding [14, 15, 16]. Most studies have therefore focused on improving processability and mitigating interfacial weaknesses through remelting, heat treatments, or interlayer addition [17, 18, 19]. However, understanding the underlying mechanisms of additive manufacturing—melt pool dynamics, keyhole behavior, bubble formation, and elemental mixing—requires time‐resolved analysis. In situ diffraction or imaging experiments at neutron, synchrotron, and free electron laser facilities have proven to be powerful tools for probing single‐material systems processed via AM, revealing phase transformations, stress and crystal defect evolution, melt pool dynamics, and crack formation, among other phenomena [20, 21, 22, 23, 24, 25]. Yet, their application to multi‐material systems remains scarce. In the Cu–Ni system, in situ alloying by LPBF enabled full‐composition‐gradient structures with corresponding changes in texture and functional properties [26]. Dual‐feedstock LPBF of Ti–Al further revealed composition‐dependent phase formation and cracking behavior [27], while controlled local mixing in Ti–316L demonstrated that spatial concentration modulation can be used to tailor phase stability and mechanical response [28]. More recent in situ and operando studies have also begun to probe phase evolution, melt‐pool mixing, and defect‐related dynamics in dissimilar‐material additive manufacturing systems [29, 30, 31, 32]. However, direct real‐time quantification of how inter‐material mixing governs transient local composition, keyhole stability, and pore formation in multi‐material LPBF is still limited. Moreover, experimental studies are constrained by material‐dependent imaging contrast and by the difficulty of resolving local composition fields during rapid melt‐pool evolution, especially for systems with similar X‐ray attenuation. For this reason, high‐fidelity numerical models are important not only as a complement to in situ experiments but also as a necessary tool for testing mechanisms that cannot be isolated directly from experiments alone.

High‐fidelity process modeling has been used in elucidating LPBF physics, from melt‐pool flow driven by recoil pressure and Marangoni convection [33] to multi‐track, multi‐layer evolution [34], powder–melt interactions [35, 36], and free‐surface dynamics captured by meshfree particle methods [37, 38, 39]. For multi‐material LPBF, emerging multiphase formulations and compositional transport simulations have begun to investigate dissimilar‐alloy interactions [40, 41, 42]. Nonetheless, these models are typically validated only qualitatively against ex situ microstructure images and lack benchmarking against time‐resolved in situ measurements. Even studies that compare Computational Fluid Dynamics (CFD) predictions with synchrotron imaging have not quantitatively resolved the evolution of local composition fields [43]. Thus, the field lacks a rigorously validated, mechanistic framework capable of describing how dissimilar materials mix, how mixing perturbs melt pool stability, and how defects emerge from these interactions.

Defect formation, particularly keyhole‐induced porosity, remains a critical challenge in metal AM due to its direct impact on structural integrity and performance. Recent advances in in situ diagnostics have substantially deepened our understanding of the mechanisms governing keyhole and pore dynamics in single‐material systems. High‐speed X‐ray imaging has shown that transient melt‐pool flow and keyhole oscillations control the nucleation and evolution of keyhole porosity [44], while operando radiography in Ti‐6Al‐4V has demonstrated that pore formation can be triggered by instabilities localized at the keyhole tip [45]. Synchrotron imaging has further mapped distinct regimes of stable and unstable keyhole operation across the processing space [46].

To capture the full multiphysics complexity of melt pool behavior, experimental diagnostics have increasingly been complemented by high‐fidelity numerical modeling. Multiphysics CFD simulations have reproduced intuitively inexplicable melt pool behaviors in ceramic systems observed via operando X‐ray tomography [47]. In metallic systems, combined modeling and imaging have elucidated pore motion and elimination mechanisms in LPBF [22, 23], linked pore formation in copper laser welding to melt pool–keyhole interactions [48], and resolved keyhole fluctuations [49] and bubble dynamics [50]. Similar coupled approaches have clarified pore nucleation and growth during directed energy deposition [51].

However, all of these insights pertain exclusively to single‐material processing. In multi‐material LPBF, melt pool behavior is complicated by composition‐dependent properties (such as viscosity, surface tension, absorptivity, density, and vaporization rates) that can fundamentally reshape keyhole stability and defect formation pathways. Despite the rapidly increasing interest in multi‐material AM, no prior study has established the mechanistic link between inter‐material mixing and defect generation during LPBF.

To address these gaps, we use the Al–Cu system as a representative multi‐material LPBF case to investigate the coupling between inter‐material mixing, keyhole dynamics, and defect formation. The present study addresses the following fundamental questions:

1. How do dissimilar alloys mix during multi‐material LPBF, and how does the resulting spatiotemporal composition distribution influence pore formation and melt pool stability?
2. To what extent can high‐fidelity multiphase, multi‐material simulations reproduce experimentally observed melt pool dynamics, and what additional mechanistic insights into dissimilar alloy mixing emerge from integrating modeling with in situ observations?
3. What physical pathways govern defect nucleation and evolution during material mixing, and how can such defects be mitigated?

In this work, we address these questions using a tightly coupled experimental‐computational framework. Operando synchrotron X‐ray radiography of Al–Cu LPBF captures the real‐time evolution of keyholes, pores, and mixing fronts, while high‐fidelity multiphase, multi‐material CFD simulations performed under matched conditions reproduce and extend the observed melt pool dynamics. Through combined analysis, we quantify the spatiotemporal evolution of composition fields and defect trajectories, revealing the mechanistic coupling between multi‐material mixing and defect formation in LPBF.

## 2 Results

### 2.1 Characterization of Keyhole Dynamics and Evolution

**FIGURE 1:**
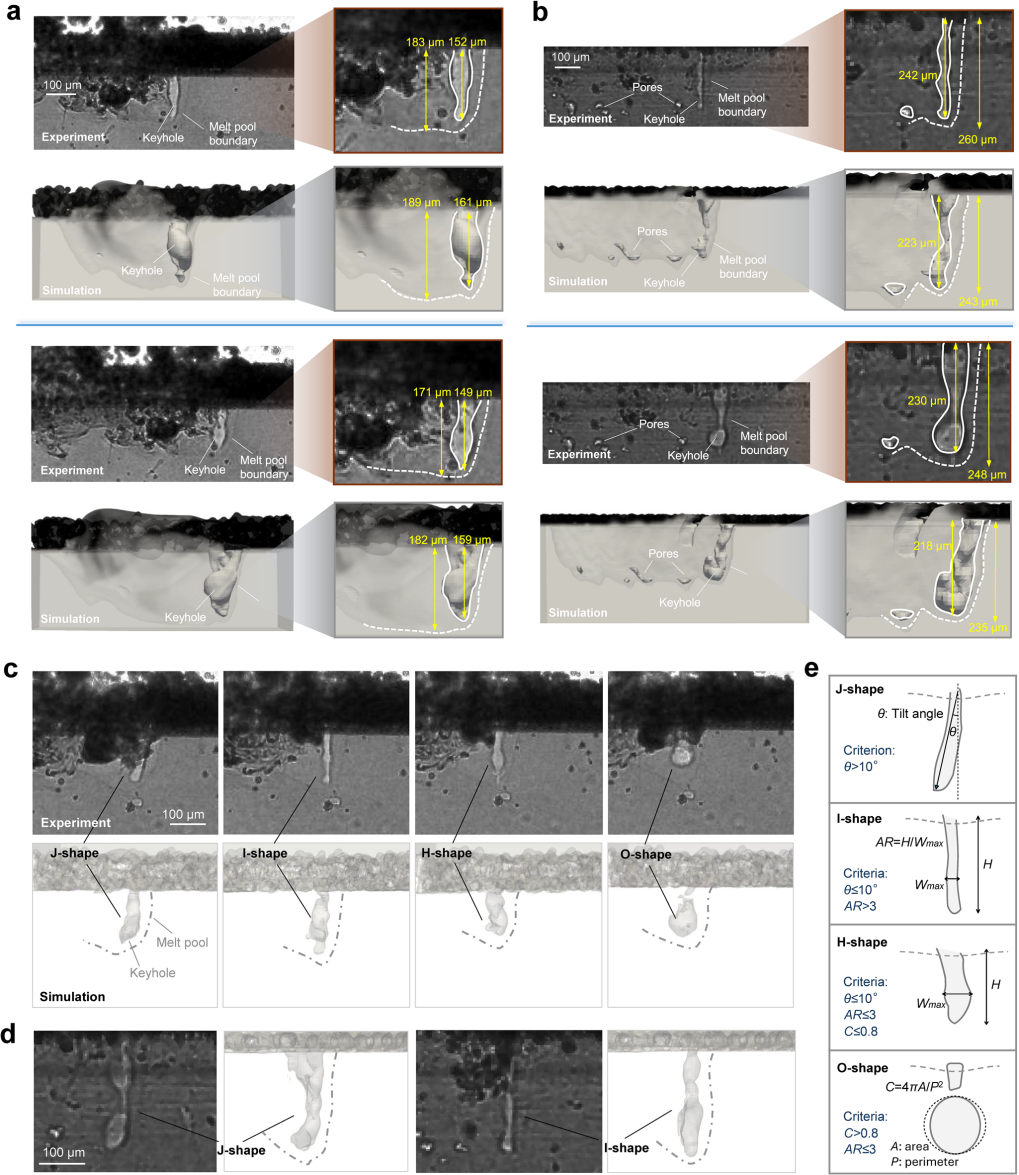
Transient keyhole morphology: synchrotron X‐ray radiography vs. high‐fidelity multiphase simulations. (a,b) Snapshots comparing experiment (top) and simulation (bottom) for powder‐bed thicknesses of (a) 150 μm and (b) 50 μm. The measured keyhole depth in the thick‐bed case is 152 μm (experiment) vs. 161 μm (simulation), 5.9 % deviation, while the thin‐bed case yields 242 μm and 223 μm, respectively (7.8 % deviation). (c) Morphological sequence for the 150 μm bed thickness. Four recurrent modes are resolved in both datasets: the keyhole *J*‐shape forms, stretches into an *I*‐shape, contracts to an *H*‐shape, and finally widens into an *O*‐shape before the cycle restarts. (d) Mode sequence for the 50 μm bed thickness. Only two shapes—*J* and *I*—are observed, oscillating periodically without transitioning to the *H* or *O* configurations seen in the thicker bed. (e) Schematic illustration of the operational geometric criteria used to distinguish the *J*‐, *I*‐, *H*‐, and *O*‐shaped keyhole morphologies.

**FIGURE 2:**
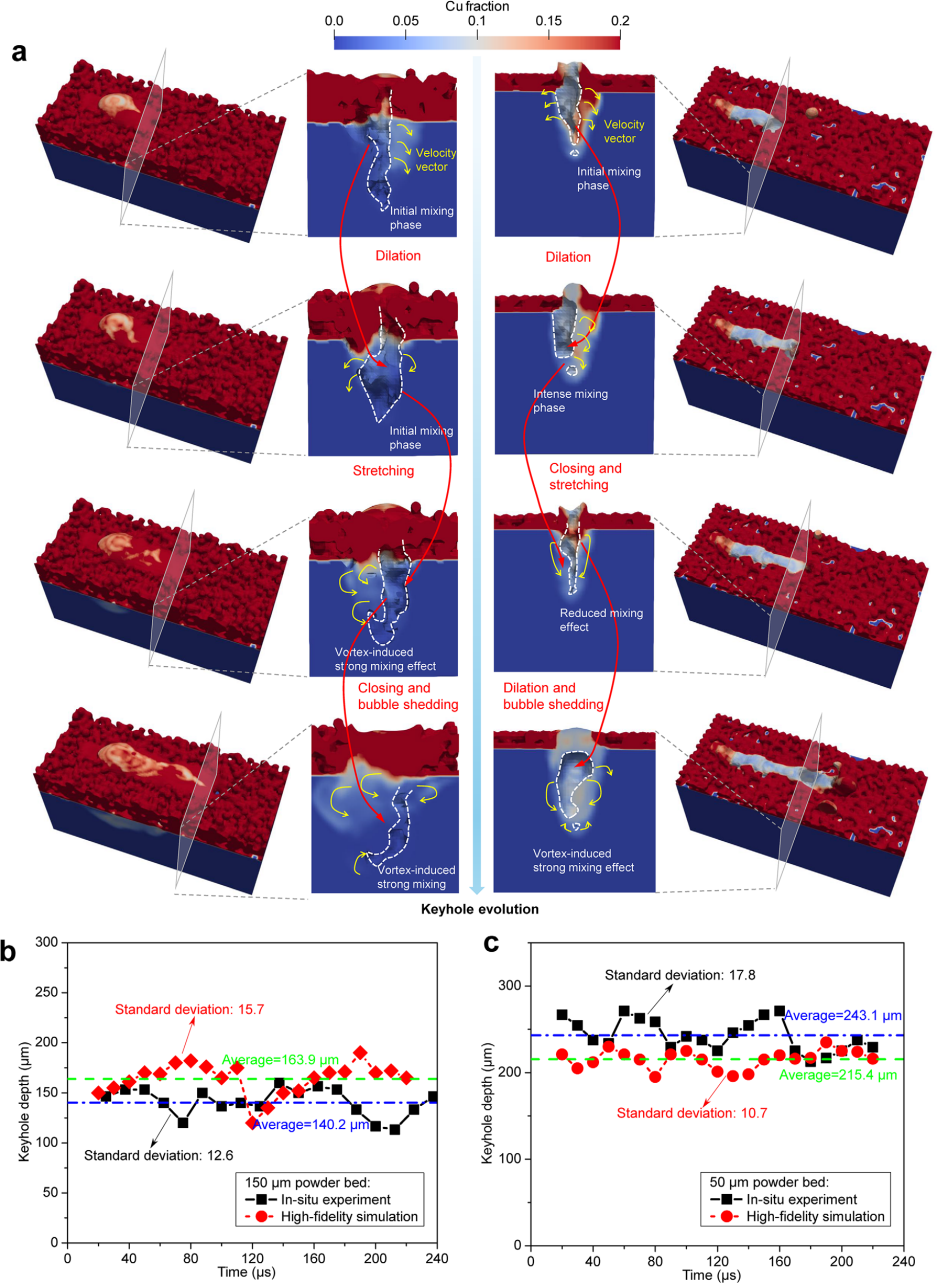
Keyhole dynamics, material mixing mechanisms, and depth evolution in multi‐material laser processing. (a) Time‐resolved snapshots of keyhole behavior and Cu–base metal mixing for powder bed thicknesses of 150 μm (left) and 50 μm (right). In the 150 μm bed, the keyhole undergoes a sequence of dilation, stretching, and eventual closure at the base, accompanied by strong vortex‐driven convection and pronounced material mixing. In contrast, the 50 μm bed exhibits weaker convection, reduced mixing, and more frequent bubble shedding, aligning with the simpler and more stable keyhole modes observed from the side. (b,c) Time‐series measurements of keyhole depth from synchrotron X‐ray imaging (black squares) and high‐fidelity multiphase simulations (red circles), for powder bed thicknesses of (b) 150 μm and (c) 50 μm. Simulations accurately reproduce both average depth and fluctuation trends, supporting the predictive fidelity of the multiphase model.

To assess the predictive fidelity of the multiphysics simulation framework, we performed a direct, time‐resolved comparison with in situ synchrotron X‐ray imaging for two representative cases under varying powder‐bed thicknesses. The comparison between experiments and simulations was not based on a strict one‐to‐one synchronization from the same absolute starting time, which is challenging in operando X‐ray observations. Instead, the comparison was performed after the keyhole had entered a stable evolution regime in both datasets. In this regime, the keyhole exhibits recurrent characteristic morphologies, enabling a physically meaningful comparison of representative states and overall fluctuation behavior. Accordingly, the comparison in Figure 1 is based on representative morphologies within the stable oscillatory regime. In Figure 2, the purpose is to provide an overall quantitative comparison of keyhole‐depth evolution after the process has reached the stable regime. Since the keyhole dynamics are already recurrent and statistically stable at this stage, the comparison does not require identical absolute starting times; instead, the relevant metrics are the agreement in mean depth, oscillation amplitude, and fluctuation trend.

The radiographs and corresponding simulation snapshots for 150 and 50 μm powder beds shown in Figure 1a,b reveal excellent agreement in both overall geometry and transient keyhole morphology. In the 150 μm case, the experimentally measured keyhole depth is 152 μm, which closely matches the simulated value of 161 μm (5.9% deviation). A similarly small discrepancy is observed for the 50 μm case (242 μm vs. 223 μm; 7.8%), revealing the quantitative accuracy of the model across disparate powder‐bed conditions.

Beyond instantaneous morphological agreement, the simulations reproduce the full temporal evolution of the keyhole. Figure 1e shows a schematic of different keyhole morphologies, which are now characterized using three geometric descriptors: (i) the tilt angle θ, defined as the angle between the build direction and the line connecting the center of the keyhole opening at the powder‐bed surface to the deepest point of the cavity; (ii) the aspect ratio *AR* = *H*/$W_{max}$, where *H* is the keyhole depth and $W_{max}$ is the maximum keyhole width; and (iii) the circularity $C=4\pi A/P^{2}$, where *A* and *P* are the contour area and perimeter, respectively. Based on these descriptors, the four morphologies are operationally defined as follows. A *J*‐shape corresponds to a tilted cavity with $\theta >10^{\circ }$. An *I*‐shape corresponds to a nearly vertical and slender cavity with $\theta \leq 10^{\circ }$ and AR>3.0. An *H*‐shape corresponds to a nearly vertical but relatively compact cavity with $\theta \leq 10^{\circ }$, AR≤3.0, and C≤0.8. An *O*‐shape corresponds to a near‐circular bulb‐like cavity with C>0.8 and AR≤3.0.

As shown in Figure 1c, the 150 μm powder bed exhibits a cyclic sequence of four recurrent configurations: a shallow *J*‐shaped cavity that progressively elongates into an *I*‐shape, contracts into an H‐shape with a narrowed midsection, and finally broadens near the surface to form an O‐shape. The model captures not only the geometry of these modes but also their characteristic ordering and periodicity. This agreement reflects the simulation's ability to resolve the coupled multiphase phenomena governing keyhole behavior, including vapor recoil pressure, melt flow recirculation, capillarity, and interface instabilities.

In contrast, the 50 μm powder bed exhibits markedly simpler dynamics (Figure 1d). Only two shapes (J and I) emerge, oscillating with a regular periodicity and without transitions to the more complex H or O morphologies. This restricted mode set suggests that, under the thinner‐bed condition, melt‐pool penetration is reduced and the deeper instabilities associated with higher‐order keyhole shapes are less pronounced. These observations are consistent with a reduced effective absorption depth and modified vapor‐driven flow, and highlight the sensitivity of keyhole dynamics to powder‐bed geometry. The corresponding real‐time evolution is provided in Videos S1 and S2. In particular, Video S1 (frames 426–450) clearly shows the full *J*‐to‐*O* transition for the 150 μm powder‐bed case, whereas Video S2 shows that only the J‐ and I‐shaped keyholes appear in the 50 μm case.

We further examined the mechanisms governing keyhole evolution in multi‐material laser processing by analyzing front‐view dynamics and mixing behavior for two representative powder bed thicknesses, 150 and 50 μm. As shown in Figure 2a, the thicker powder bed exhibits a richer sequence of keyhole transformations, driven by the interplay of recoil pressure, fluid flow, and composition‐dependent thermophysical response.

In the 150 μm case, the keyhole initially forms as a narrow channel that dilates and stretches as vapor recoil and capillary forces trigger vigorous melt‐pool convection. Vortical structures emerge along the keyhole walls and promote substantial mixing between Cu and the base metal, yielding a visibly broader melt‐pool cross‐section. As the process continues, the base of the keyhole begins to close, often accompanied by bubble shedding from the lower cavity. These dynamics correspond to the *J*‐to‐*O* transition seen in side‐view imaging and reflect a transient but highly repeatable sequence of fluid‐driven events.

The thinner powder bed (50 μm) follows a more restrained trajectory. Although the keyhole still undergoes phases of dilation and partial closure, convection is weaker and Cu mixing remains localized. The melt pool stays comparatively narrow, and bubble shedding occurs more frequently and earlier in the process. Under the thinner‐bed condition studied here, the melt pool shows weaker convection and reduced large‐scale flow development, leading to simpler keyhole modes and limited lateral growth.

The depth measurements in Figure 2b,c quantify these differences. While the mean keyhole depths differ only moderately, the thicker bed exhibits stronger depth oscillations, consistent with enhanced internal flow and intermittent collapse events. In contrast, fluctuations in the 50 μm case are substantially dampened, mirroring the reduced mixing activity in the front‐view sequences.

Collectively, these observations underscore that powder bed thickness strongly influences the thermal and hydrodynamic state of the melt pool. Energy absorption, convection intensity, material mixing, and keyhole stability are tightly coupled, providing opportunities to tailor compositional gradients and microstructure in multi‐material additive manufacturing.

### 2.2 Multiscale Analysis of Pore Formation Mechanisms

**FIGURE 3:**
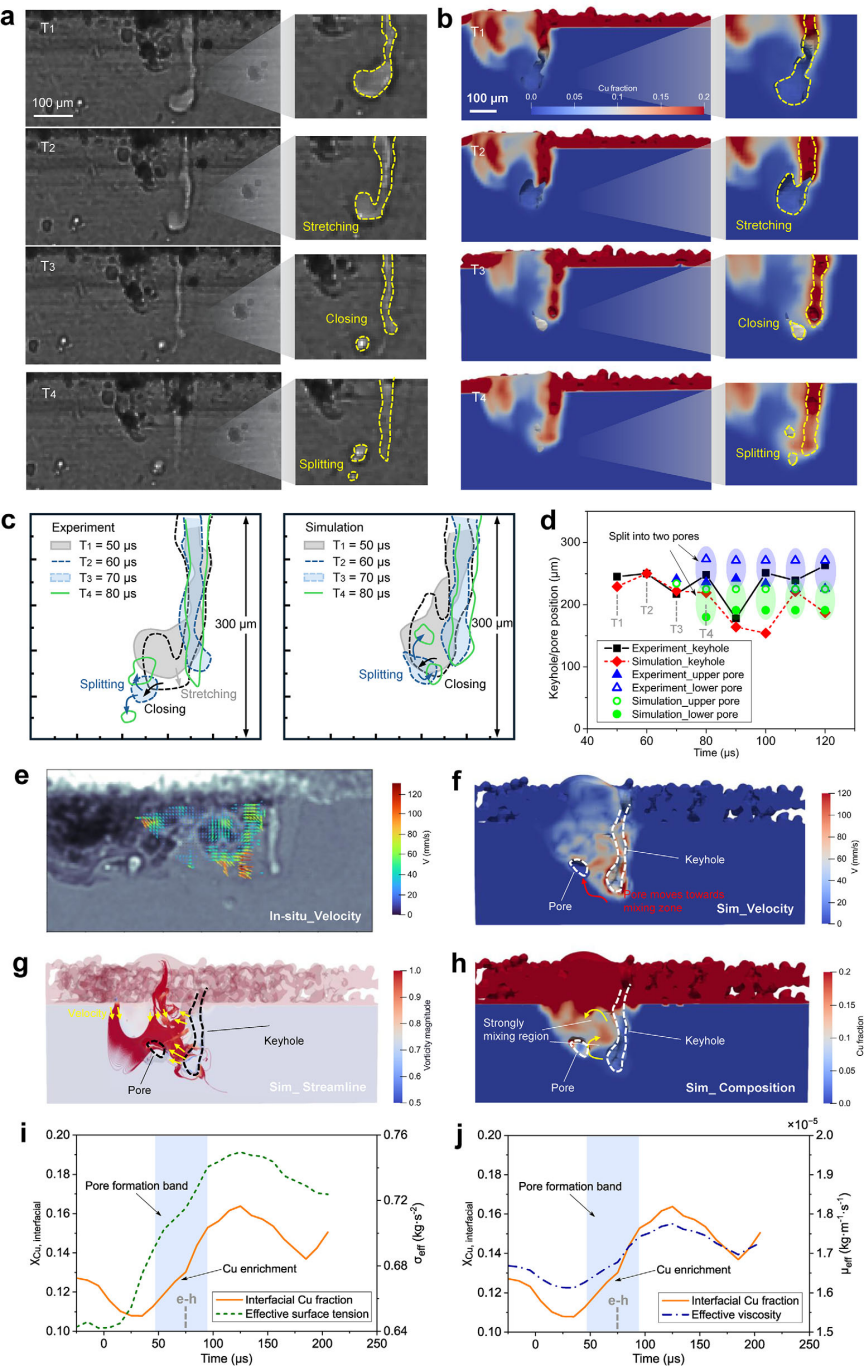
Formation and evolution of large pores during the keyhole collapse. (a,b) Time‐resolved sequences showing the formation of a large pore due to keyhole stretching and closure, as captured by (a) in situ X‐ray imaging and (b) multiphase simulation. (c) Overlay of keyhole and pore profiles at successive time points ($T_{1}$–$T_{4}$), illustrating the pore formation and splitting process in both experiment (left) and simulation (right). (d) Quantitative tracking of keyhole depth and pore positions over time. (e) In situ velocity field measured within the melt pool, showing lateral flow near the keyhole. (f) Simulated velocity field revealing high‐velocity domains and recirculating flow patterns near the pore. (g) Simulated streamlines indicate strong vortex activity behind the keyhole, which drives the upward motion of the large pore. (h) Simulated material composition field showing a region of intense mixing in the same area, further explaining the trajectory and behavior of the pore. (i,j) Time evolution of composition‐dependent interfacial properties in the selected interfacial band near the rear keyhole wall during the same large‐pore event. (i) Time evolution of the interfacial Cu fraction and effective interfacial surface tension. (j) Time evolution of the interfacial Cu fraction and effective interfacial viscosity.

To determine the origin of large pores observed in multi‐material LPBF, we analyzed the transient dynamics of keyhole collapse using synchronized in situ X‐ray imaging and multiphase simulation. Figure 3a,b shows that large pores emerge via a characteristic collapse sequence: the keyhole first elongates vertically, then closes at its base, trapping a vapor cavity that evolves into a distinct pore. Within a few microseconds, this cavity splits into two daughter pores (i.e., bubbles) migrating in opposite directions. This process can also be directly observed in Video S2 over the interval from 2.55 to 2.62 ms.

Temporal snapshots ($T_{1}$‐$T_{4}$) captured in experiment and simulation consistently show cavity stretching, necking, pinch‐off, and final bifurcation, with yellow contours marking the gas‐liquid interface and narrowing throat where detachment initiates. The overlay in Figure 3c emphasizes the near‐identical evolution of keyhole and pore profiles in both datasets. Quantitative tracking (Figure 3d) confirms this correspondence: keyhole depth and the trajectories of the upper and lower daughter pores closely match between experiment and simulation. The upper pore moves toward the melt surface, while the lower pore remains near the keyhole tip.

To elucidate the forces driving this motion, we examined nearby melt‐pool flow fields. In situ velocimetry (Figure 3e) reveals lateral and upward flow behind the keyhole, suggesting a convective contribution. Simulations (Figure 3f) show high‐velocity regions surrounding the detached pore, while streamlines (Figure 3g) reveal a strong trailing‐edge vortex imparting upward momentum to the upper pore. The composition field (Figure 3h) identifies a zone of intense Cu mixing co‐located with this vortex, indicating that the same convective mechanisms governing alloy transport also control pore displacement.

To quantify how the local composition field feeds back on keyhole collapse, we extracted composition‐dependent interfacial properties in a selected interfacial band near the rear keyhole wall, i.e., the region where the large pore subsequently forms. The sampling band was defined within a local box around the rear‐wall mixing zone and restricted to interfacial cells in which the gas fraction and metallic fraction simultaneously indicate a metal–gas boundary region. The selected interfacial band satisfies $0.05<\alpha_{N}<0.4$, $\alpha_{m}>0.55$, and γ>0.1, where $\alpha_{N}$ is the gas volume fraction, $\alpha_{m}$ is the total metallic volume fraction, and γ is the liquid fraction. $\chi_{\mathrm{Cu}}$ denotes the Cu fraction averaged over the selected interfacial band near the rear keyhole wall. The effective surface tension was calculated as the local composition‐weighted average of the Cu–gas and Al–gas surface tensions. The effective interfacial viscosity was evaluated in the same manner.

As shown in Figure 3i,j, the interfacial Cu fraction increases over the pore formation band and is accompanied by increases in both the effective interfacial surface tension and the effective interfacial viscosity. These results provide direct quantitative evidence that the composition evolution raises the local surface‐tension coefficient and viscosity in the region where the vapor cavity subsequently necks and pinches off. Importantly, this result should not be interpreted as implying that increasing Cu content alone directly triggers instability. Rather, the quantitative trends in Figure 3i,j show that Cu enrichment alters the local force balance governing keyhole collapse by modifying the interfacial properties entering the momentum equation. The large pore is then generated through the coupled action of recoil pressure, composition‐dependent interfacial‐property variation, and the evolving melt‐flow topology, which is consistent with the collapse sequence and vortex‐assisted pore transport resolved in Figure 3a–h.

### 2.3 Mechanisms Governing the Formation of Small Pores

Beyond large pores, in situ observations and simulations reveal two distinct classes of small pores, i.e., Type I and Type II, originating from separate mechanisms within the melt pool.

**FIGURE 4:**
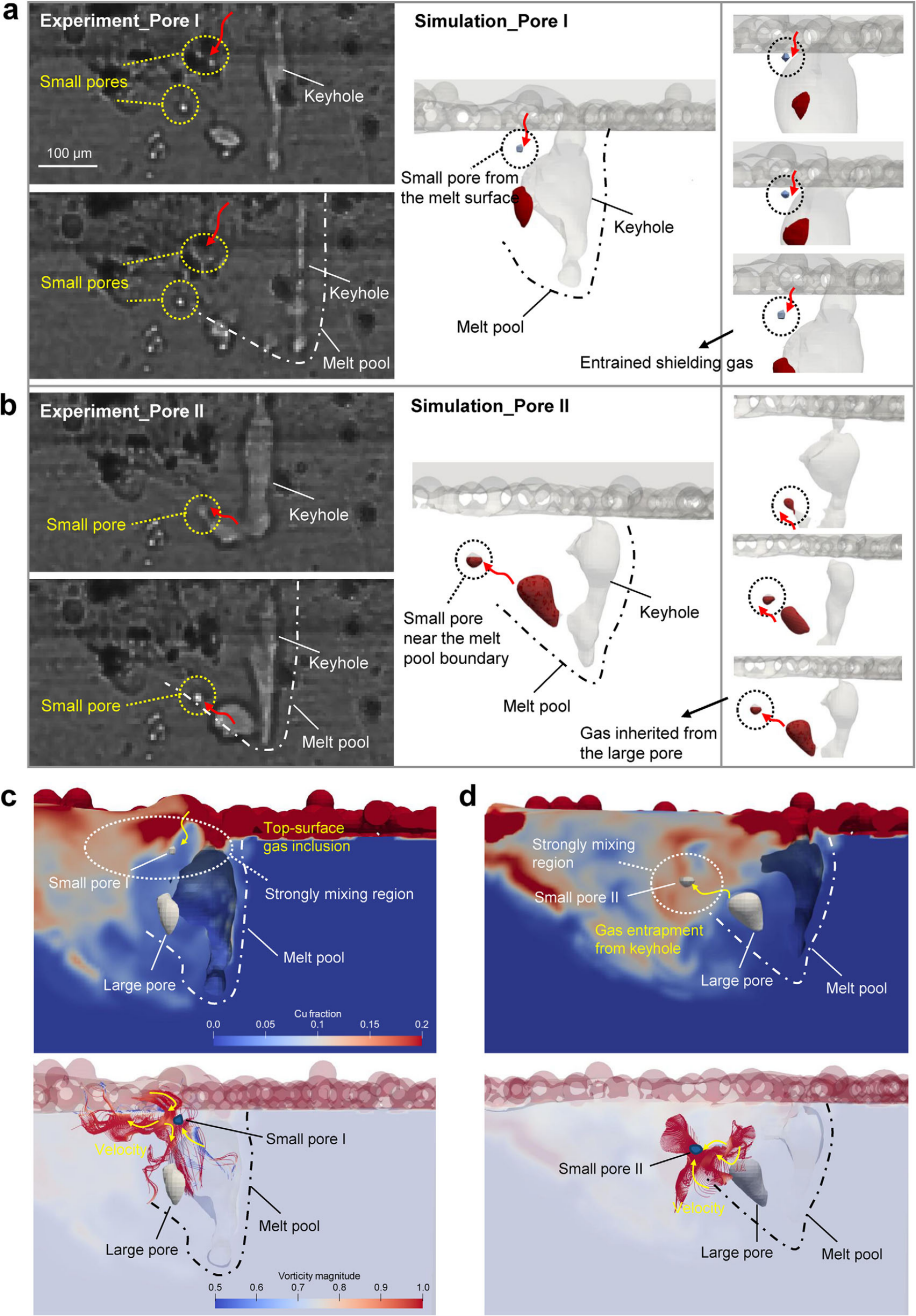
Mechanisms governing the formation of small pores. (a) Time‐resolved in situ radiographs (left) and corresponding multiphysics simulation (right) showing Type I small pore formation. Gas‐origin tracking indicates that this pore originates from entrained shielding gas (blue) above the melt surface. (b) Formation of Type II small pores captured in both the experiment (left) and simulation (right). Unlike Type I, this pore forms below the free surface as a daughter pore detached from a large pore during partial collapse near the melt‐pool boundary. Gas‐origin tracking shows that the gas inside the Type II pore is inherited from the large pore (red). (c) Simulation results showing the Cu composition field (top) and vorticity streamlines (bottom) during Type I pore formation. Surface flow induced by Marangoni convection drives shielding gas into a high‐vorticity mixing zone, where it becomes trapped and forms a small pore. (d) Cu composition (top) and streamlines (bottom) during Type II pore formation. The small pore detaches from a larger one and is carried into a strongly mixed region near the melt pool boundary.

Figure 4a shows a Type I small pore that first appears near the melt pool surface and is subsequently drawn into the melt pool by surface‐directed flow. Gas‐origin tracking in the simulation shows that this pore forms by entrainment of shielding gas from above the melt surface. In contrast, Type II pores (Figure 4b) originate from the subsurface, forming as secondary bubbles that pinch off during the partial collapse of a larger pore. The gas tracking in the simulation confirms that the gas inside the Type II pore is inherited from the large pore.

Figure 4c,d reveals the flow structures responsible for these behaviors. During Type I formation (Figure 4c), the composition field shows a strongly mixed region just below the surface, with steep Cu concentration gradients generated by Marangoni stresses. Streamlines highlight surface‐driven vortices producing a downward suction that traps surface gas pockets as small pores. For Type II (Figure 4d), pore formation occurs in a region dominated by high vorticity between a collapsing large pore and the melt pool boundary. This strong rotational flow promotes daughter‐pore detachment and guides its migration outward. The formation of Type I and Type II small pores is also directly captured in Video S2 over 2.59–2.64 ms and 2.82–2.86 ms, respectively.

These findings demonstrate that small pore formation is not solely tied to powder packing or surface roughness. Instead, pores arise dynamically from local melt‐flow instabilities, composition gradients, and gas–liquid interactions. Whether such pores become trapped, merge, or escape depends sensitively on the surrounding vortex structures, which, in turn, highlights the need for process strategies that modulate convection, shielding gas, and alloy composition to mitigate porosity in multi‐material LPBF.

### 2.4 Quantitative Assessment of Defect Formation and Composition Evolution

**FIGURE 5:**
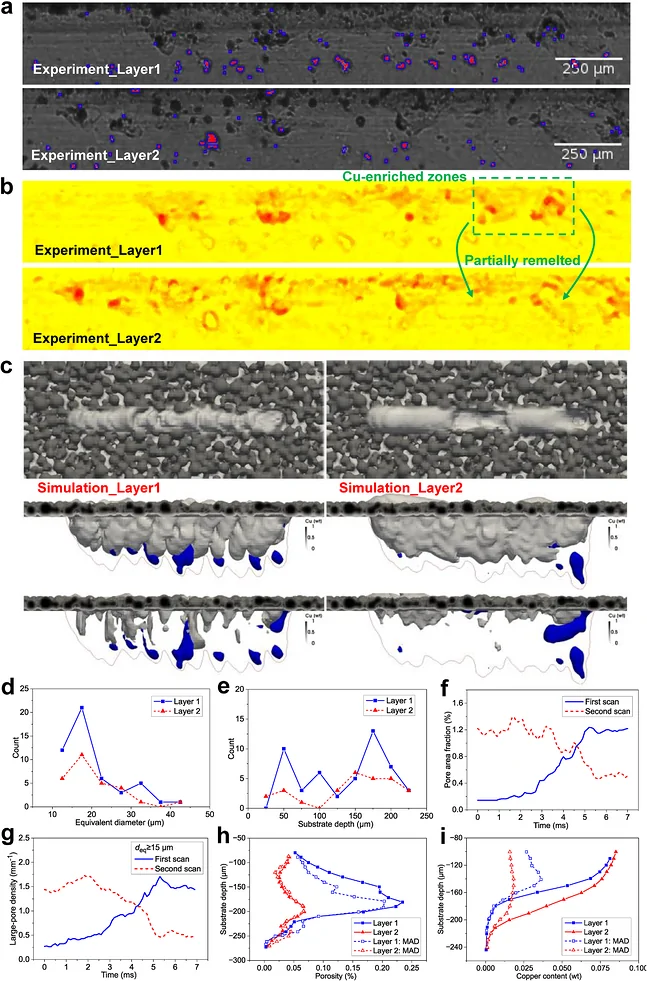
Porosity, copper distribution, and pore statistics across two successive layers. (a) In situ X‐ray images after the first (top) and second (bottom) scans; detected pores are outlined. (b) Post‐processed Cu maps for the same layers. The second scan partially remelts and redistributes the inherited Cu‐rich regions, together with additional Cu entrainment and convective transport. (c) Simulated melt tracks for the first (left) and second (right) scans. The second pass smooths the Cu distribution and limits deep, high‐concentration pockets. (d) Pore‐count distribution vs. equivalent diameter. (e) Depth‐resolved pore‐count distribution. (f,g) Time‐resolved pore evolution during the first and second scans: (f) projected pore area fraction, and (g) density of large pores. (h,i) Depth‐resolved comparisons between the two layers obtained from simulations: (h) porosity profiles, and (i) Cu‐content profiles.

We next quantified how defect populations and Cu distribution evolve across two successive scans performed under identical conditions (12.1 J/${\mathrm{mm}}^{2}$, 50 μm powder bed, 300 K boundary temperature). The in situ images in Figure 5a show that pores generated during the first pass accumulate primarily near the bottom of the melt track. Following the second pass, many of these voids are either removed or fragmented and displaced toward mid‐depth, consistent with renewed melting and fluid recirculation.

Composition maps (Figure 5b) and depth‐resolved profiles (Figure 5i) reveal a clear increase in Cu content after the second scan. First, previously formed Cu‐rich regions are remelted and reincorporated into the liquid pool. Second, remelting entrains additional Cu from the fresh powder layer, increasing the total solute available for mixing. Meanwhile, convection is intensified during the second pass, driven by strengthened recoil pressure and Marangoni shear acting along the enlarged melt surface, which redistributes both the inherited and newly entrained Cu more efficiently throughout the remelted pool. These effects promote deeper and more uniform mixing, yielding a smoother Cu profile across nearly the entire melt depth.

To further quantify the effect of rescanning on pore evolution, additional image‐based pore statistics were extracted from the experimental radiographs. As shown in Figure 5d, the pore‐count distribution vs. equivalent diameter indicates that the second scan reduces the overall pore population, with the reduction being more pronounced for the large‐pore population. Figure 5e shows that the pore‐count distribution along the substrate depth also changes markedly after rescanning: the deeper pore‐rich region is substantially reduced, whereas only a smaller residual pore population remains in other regions.

During the first scan, both the projected pore area fraction in the pore‐rich region and the density of large pores increase with time (Figure 5f,g), indicating progressive pore accumulation during the initial melting process. In contrast, during the second scan, both quantities decrease progressively, consistent with pore shrinkage and elimination during remelting. Combined with the experimentally observed partial remelting of the Cu‐enriched region and the remelting behavior reproduced in the simulations, these results indicate that the second scan reactivates melt‐pool convection and partially remelts the pore‐rich zone, thereby promoting pore shrinkage, breakup, transport, and escape. Pores that remain after rescanning are therefore likely those located in deeper regions or in local weak‐flow regions, where the time available for pore elimination before solidification is limited.

Simulations (Figure 5c) further capture these trends: after the first scan, discrete Cu‐rich pockets extend into the substrate, whereas the second pass significantly reduces sharp concentration gradients and produces a more continuous distribution of Cu. Enhanced mixing also contributes to the reduction of mid‐depth porosity (Figure 5h), as buoyancy‐driven recirculation and repeated interface renewal facilitate bubble breakup and escape.

Together, these results imply that the second scan acts simultaneously as a remelting step, a homogenization step, and a means of introducing additional Cu into the melt pool. It redistributes the Cu inherited from the previous layer, incorporates additional Cu from the new powder, and removes a substantial fraction of mid‐depth pores. The outcome is a modest but beneficial increase in overall Cu content while maintaining reduced porosity through the core of the track.

## 3 Discussion

In this work, we combined operando synchrotron X‐ray radiography with high‐fidelity multiphysics modeling to directly observe, in real time, how keyholes, melt‐pool mixing, and pore populations evolve in a representative multi‐material LPBF system. Whereas most prior studies have focused on single‐alloy processing, our results show that introducing a second material with distinct thermophysical properties fundamentally modifies both keyhole dynamics and the mechanisms governing defect formation. Compared with earlier multi‐material LPBF models [41], the present work does not primarily seek a more general numerical framework, but rather a quantitatively benchmarked mechanistic understanding of defect formation in a dissimilar‐alloy system. Earlier numerical studies mainly emphasized modeling capability, and their validation was typically based on ex situ observations or literature‐level comparisons rather than time‐resolved operando measurements. As a result, the roles of inter‐material mixing in defect formation and transient composition evolution can be discussed directly in the present study.

A central outcome is the link we establish between multi‐material mixing and the sequence of keyhole morphologies. For thicker powder beds, the keyhole progresses through a rich set of modes that appear as *J*‐, *I*‐, *H*‐, and *O*‐shaped cavities from the side view. Front‐view modeling reveals that these shapes correspond to alternating phases of dilation, stretching, and partial closure of the vapor cavity, driven by recoil pressure, Marangoni flow, and buoyancy. Strong vortices develop behind the keyhole and within the compositional boundary layer, creating intense Cu–Al mixing and large transient fluctuations in keyhole depth. Reducing powder bed thickness suppresses much of this behavior: the flow field weakens, the mixing zone shrinks, and keyhole dynamics become markedly simpler. Thus, even modest geometric variations in the powder bed can qualitatively reshape multi‐material melt pool dynamics.

Our second major finding concerns the formation and transport of pores of different sizes. Large pores originate when a vertically stretched keyhole closes from below, entrapping a vapor cavity that subsequently pinches off and splits into two daughter pores. Time‐resolved reconstructions show that this process is governed by composition‐driven flow instabilities: as Cu‐rich and Al‐rich melts interpenetrate, the local flow becomes highly vortical, the rear keyhole wall oscillates, and a necked vapor ligament forms and ruptures. After detachment, the upper daughter pore is advected upward by the trailing‐edge vortex, while the lower pore remains near the keyhole root. These observations indicate that keyhole porosity in multi‐material systems is governed not only by classical power–velocity combinations but also by composition‐dependent differences in surface tension, density, and viscosity, which modulate the onset and geometry of collapse.

In addition to large pores, we identify two distinct families of small pores that originate from different gas‐entrainment routes. Type I pores originate at the melt surface, where Marangoni‐driven surface flow and shielding‐gas shear create a high‐vorticity mixing layer that draws gas pockets into the melt pool. Type II pores form instead by the breakup of pre‐existing large pores, typically near the melt pool boundary. High local vorticity facilitates the detachment of these daughter bubbles and directs their migration along the pool periphery. Both mechanisms highlight the central role of the evolving flow topology and composition gradients in determining where and how small pores emerge. Accordingly, strategies that modulate surface flow, adjust scan strategies, or tailor shielding‐gas conditions may effectively suppress fine‐scale porosity in multi‐material builds.

These results also help clarify how the present study differs from earlier experimental work on multi‐material LPBF. For example, Gaudez et al. [52] used a similar radiographic approach to study material redistribution in an Al–Cu system and showed that Cu incorporation occurs through discrete stochastic events, which in turn affect local solidification paths and the resulting microstructure. The emphasis there was on redistribution and microstructure formation. Here, by contrast, the main advance is that inter‐material mixing is directly connected to keyhole‐shaped transitions and pore formation pathways, and these relationships are further interpreted through quantitatively benchmarked simulations. This makes it possible to discuss not only how the second material is redistributed, but also how that redistribution changes melt‐pool stability and defect generation in real time.

The present results also suggest that powder‐bed thickness should be considered as an additional process variable in multi‐material LPBF. In single‐material systems, powder layer is commonly discussed in terms of its influence on melt‐pool and pore dynamics [53]. In the present multi‐material system, however, powder‐bed thickness is also associated with the degree of inter‐material mixing, and may therefore influence the local thermophysical response in addition to energy absorption and melt‐pool geometry. This helps explain why the thicker powder bed in the present study exhibits stronger mixing, richer keyhole mode transitions, and more pronounced instability, whereas the thinner bed shows simpler keyhole dynamics and weaker mixing. Powder‐bed thickness in multi‐material LPBF, therefore, acts not only as a geometric parameter but also as a practical process variable influencing composition‐dependent keyhole regulation.

The layer‐wise analysis shows that repeated scanning simultaneously alters defect populations and material composition. After the first scan, pores accumulate near the bottom of the track, and Cu‐rich channels extend into the substrate. The second scan remelts this region, redistributes solute, and reduces porosity in the mid‐depth zone. However, it also increases the overall Cu content and smooths the vertical composition profile through enhanced convective transport. This reveals an inherent trade‐off: rescanning can homogenize composition and mitigate porosity, but it also drives additional solute transport into the substrate and modifies the final compositional gradient. In the context of functionally graded materials, this trade‐off can be exploited to tailor both defect levels and chemical profiles via the careful design of scan sequences, energy densities, and layer strategies. Recent studies further indicate that process design in multi‐material LPBF also depends on material arrangement, deposition strategy, and the design of vertical material interfaces. For example, work on intralayer deposition of dissimilar materials and on staggered multi‐material heterostructures shows that local deposition sequence and interfacial design can strongly affect build quality and final properties [54, 55]. In this context, the present results highlight the importance of considering scan strategy and remelting conditions together with material layout and interface design in multi‐material LPBF.

The operando radiography furnishes quantitative, time‐resolved measurements of keyhole and pore trajectories, while the simulations resolve the underlying temperature, velocity, vorticity, and composition fields that remain inaccessible experimentally. Together, these tools reveal that defect formation in multi‐material systems emerges from a competition between recoil‐driven keyhole opening, composition‐dependent interfacial tension, buoyancy, and the dynamically evolving flow topology within the mixing zone. This framework explains why small variations in powder bed geometry, scan history, or alloy pairing can lead to markedly different melt pool behaviors and defect populations.

Some limitations should be acknowledged. The present study focuses on a single alloy pairing and a limited parameter space, and the simulations neglect certain microscopic processes—such as solidification structure, gas chemistry, and powder‐level heterogeneity—that may influence defect formation in other systems. Nevertheless, the mechanisms identified here should be interpreted within the scope of the present Cu–Al system and, more generally, for multi‐material LPBF conditions in which the constituent melts can interpenetrate and develop strong local thermophysical‐property gradients. Under such conditions, mixing‐induced changes in surface tension, density, viscosity, and absorptivity can modify melt‐pool flow topology, keyhole stability, and pore dynamics in a broadly relevant way. By contrast, systems with poor mutual miscibility, strong chemical reactivity, and rapid intermetallic‐compound formation may exhibit qualitatively different interface dynamics, mixing patterns, and defect pathways. Future work should therefore extend this framework to a wider range of alloy systems and establish how the balance between broadly relevant hydrodynamic mechanisms and system‐specific metallurgical effects governs defect formation.

## 4 Conclusion

In conclusion, this study establishes a validated experimental–computational framework for analyzing defect formation and composition evolution in the Cu–Al multi‐material LPBF. By combining operando synchrotron X‐ray radiography with high‐fidelity multiphysics simulations, we identify how composition‐dependent melt dynamics control keyhole instability, pore formation, and solute redistribution during dissimilar‐alloy processing. Our findings suggest two practical directions for engineering optimization. First, reducing powder bed thickness can suppress complex keyhole oscillations and vortex‐driven instabilities, thereby lowering the likelihood of keyhole‐collapse‐induced large pores. Second, a controlled rescanning or remelting step can be used to reduce residual porosity and improve compositional homogenization by promoting pore breakup, escape, and convective solute redistribution. At the same time, this second strategy must be applied with caution, because enhanced remelting also increases solute transport into the substrate and may alter the intended compositional gradient. Process optimization in multi‐material LPBF should therefore balance defect suppression against compositional fidelity according to the target application. Overall, the present work advances the fundamental understanding of defect generation and elimination in the LPBF process, with particular emphasis on multi‐material interactions. We elucidate the physical mechanisms that link process parameters and material properties to porosity evolution. This study, therefore, provides a quantitative basis for the informed design of improved scan strategies, the expansion of viable process windows, and the fabrication of highly reliable, compositionally tailored components.

## 5 Experimental Section

### 5.1 Materials and Printing Parameters

The precursor powder and laser processing parameters used to investigate melt pool dynamics are described below.

A spherical copper‐based alloy powder supplied by m4p (Austria) was used, with a nominal chemical composition (mass %) of Cu–0.9Cr–0.07Zr and trace amounts of Fe, Si, and O. The powder size distribution was *d* 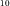 = 38 μm, *d* 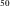 = 49 μm, and *d* 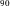 = 61 μm. The substrate material was a commercially pure aluminum plate (99.99%) with a thickness of 0.5 mm. All experiments were conducted using the miniSLM system [56], which is specifically designed for synchrotron‐based additive manufacturing studies. The high‐energy laser beam used in the miniSLM system had a wavelength of 1064 nm (redPOWER, SPI Lasers Ltd., UK).

For the thinner powder bed case, two layers were sequentially manufactured in an argon atmosphere using the following processing parameters: laser power of 200 W, scanning speed of 500 mm/s, focused laser beam diameter (1/$e^{2}$) of 33 μm, powder bed thickness of approximately 50 μm, and a unidirectional scan strategy. For the thicker powder bed, the process used a laser power of 460 W, scan speed of 400 mm/s, spot diameter of 45 μm, and a powder bed thickness of 150 μm. The reported powder‐bed thicknesses refer to values after powder spreading. To evaluate bed‐thickness uniformity, the powder‐bed height was measured at multiple locations after spreading, giving mean deviations of 8.9 and 6.1 μm for the thinner and thicker powder bed conditions, respectively.

The two parameter sets were chosen based on prior process optimization, the constraints of the operando synchrotron X‐ray imaging setup, and the requirement to achieve representative keyhole‐dominated melting conditions for each powder‐bed thickness. For the thinner powder bed, the selected condition reliably melted the Cu powder while producing a clearly resolvable keyhole and defect‐formation regime. For the thicker powder bed, the parameters were adjusted to provide sufficient energy input for melting the substantially thicker powder bed and to sustain a penetrative keyhole under the same experimental platform.

As a practical reference, the layer‐thickness‐normalized energy input remained of the same order, with values of approximately 8.0 and 7.66 J/${\mathrm{mm}}^{2}$ for the 50 and 150 μm powder‐bed cases, respectively. This normalization was used only as a practical guideline to maintain a comparable energy‐input level relative to powder‐bed thickness, rather than as a strict equivalence criterion.

### 5.2 Operando X‐Ray Radiography

**FIGURE 6:**
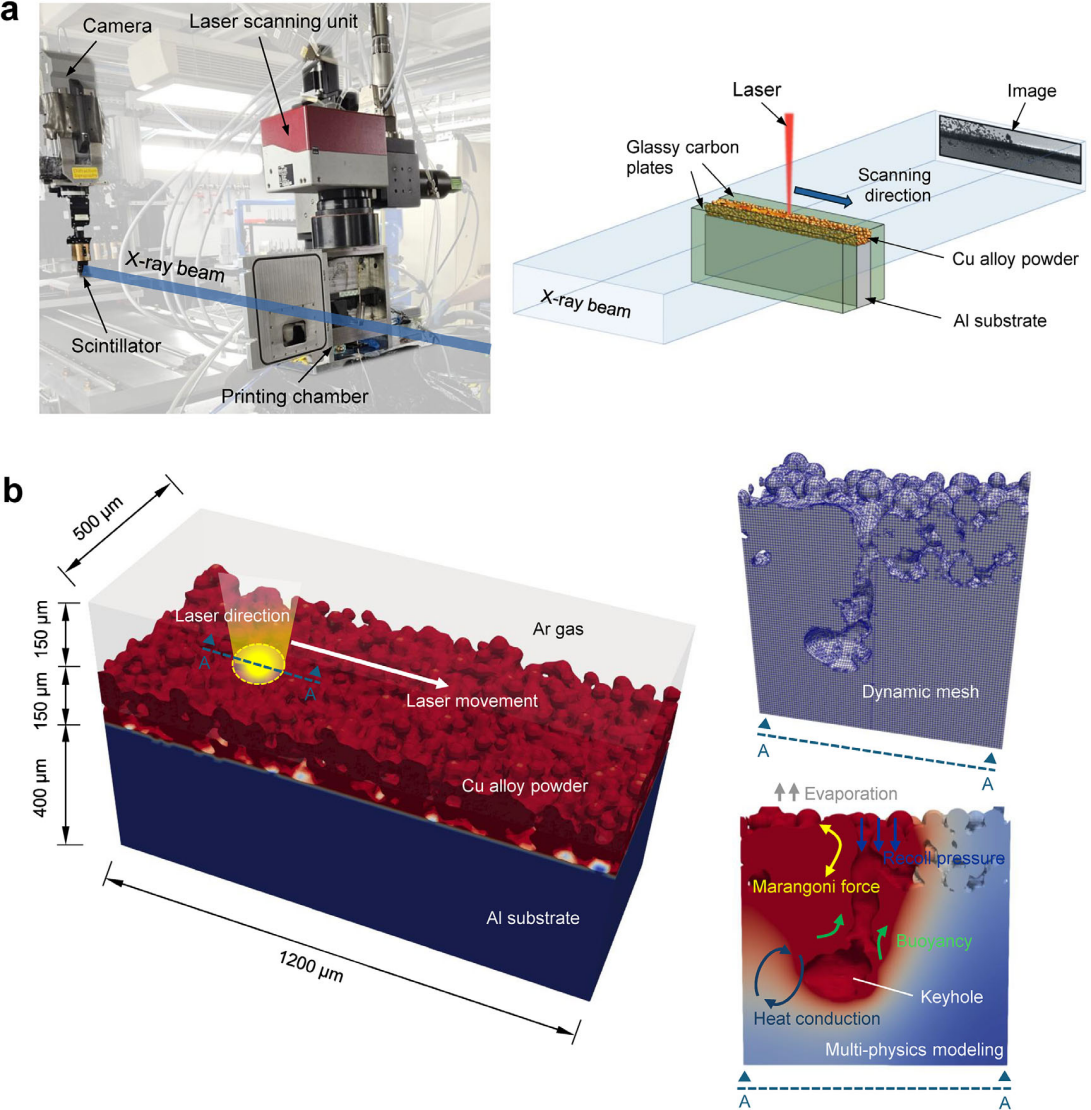
Experimental setup and numerical model for multi‐material LPBF. (a) Schematic and photograph of the in situ high‐speed synchrotron X‐ray imaging system. The system integrates a laser scanning unit, scintillator, and high‐speed camera, with the X‐ray beam passing horizontally through the printing chamber. The schematic (right) illustrates the configuration of the Cu alloy powder layer atop an Al substrate, confined between two glassy carbon plates to allow for X‐ray transmission and imaging. (b) Computational setup for the multi‐material LPBF model. The 3D domain (left) shows the distribution of Cu alloy powder on top of an Al substrate, with the laser scanning along the indicated path. The right panels show a longitudinal cross‐section revealing the dynamic mesh (top) and the associated multi‐physics processes (bottom), including evaporation, recoil pressure, Marangoni flow, buoyancy‐driven convection, and heat conduction, all of which contribute to the evolution of the keyhole and melt pool.

Operando X‐ray radiography experiments were conducted at the ID19 imaging beamline of the European Synchrotron Radiation Facility (ESRF), following the experimental configuration reported by Gaudez et al. [52]. A schematic of the setup is shown in Figure 6a.

The powder bed was confined between two glassy carbon plates mounted inside the miniSLM chamber. Diamond and beryllium windows were incorporated into the X‐ray beam path to ensure transmission. To enable high‐speed radiography, two beryllium compound refractive lenses were introduced to increase the photon flux density at the detector. The X‐ray beam had a peak energy of 19 keV, providing sufficient absorption contrast between aluminum, copper, and their mixtures.

Radiographs were acquired using a CMOS‐based high‐speed camera (Photron SAZ, Japan) coupled to a 250 μm thick LuAg:Ce single‐crystal scintillator and a 5× objective lens (numerical aperture 0.14, Mitutoyo, Japan). The resulting field of view was approximately 2.8 × 1 ${\mathrm{mm}}^{2}$, with an effective pixel size of 4.14 μm. Images were recorded at a frame rate of 100 000 frames/s during the manufacturing process. Synchronization between laser processing and image acquisition was achieved via a trigger signal generated by the miniSLM system. The movies generated from the X‐ray raw data for the thicker and thinner powder‐bed scenarios are provided in Videos S1 and S2, respectively.

### 5.3 Quantification of Cu Composition From Radiographs

Local copper composition was extracted from operando radiographs using the Beer–Lambert law combined with mixture and compound attenuation models [57], following an approach similar to that of Liotti et al. [58]. Alloying elements in the copper powder were neglected owing to their low mass fractions. The X‐ray beam was treated as monochromatic at 19 keV, and temperature‐dependent variations in mass attenuation coefficients were disregarded.

Mass attenuation coefficients at 19 keV were obtained using the XrayDB Python package, yielding values of 38.90 ${\mathrm{cm}}^{2}$/g for Cu and 3.99 ${\mathrm{cm}}^{2}$/g for Al. The Beer–Lambert inversion was based on the X‐ray mass attenuation coefficient (μ/ρ), which is generally treated as weakly dependent on physical state and temperature for practical X‐ray attenuation analysis. Under the present 19 keV monochromatic‐beam approximation, the dominant temperature‐related uncertainty in the reconstructed Cu mass fraction therefore arises from the density term, ρ(*T*).

**FIGURE 7:**
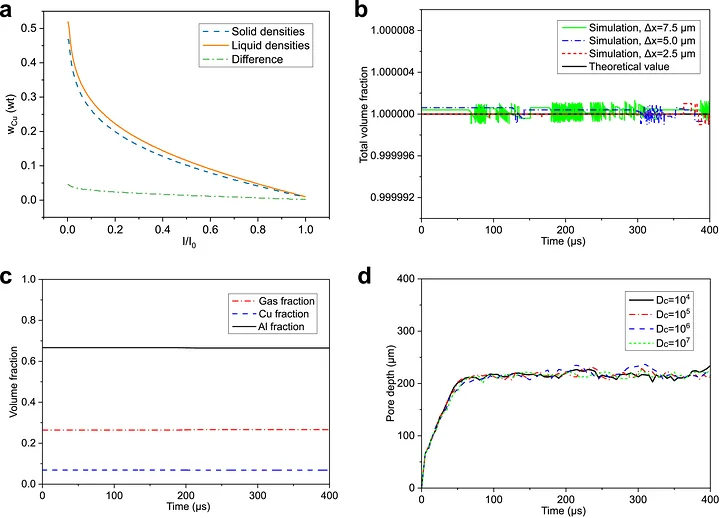
Additional validation and sensitivity analyzes for the quantitative Cu inversion and the multiphase multi‐material model. (a) Cu mass fraction as a function of the X‐ray attenuation assuming solid and liquid densities. The difference between the two curves provides a conservative upper‐bound estimate of the systematic uncertainty associated with neglecting temperature‐dependent density variations. (b) Simulated temporal evolution of the total volume fraction for three mesh sizes, Δx = 7.5, 5.0, and 2.5 μm. (c) Simulated temporal evolution of the globally integrated gas, Cu, and Al volume fractions. The nearly constant phase fractions indicate that numerical diffusion across the interfaces is very limited during the three‐phase VOF simulation. (d) Effect of the Darcy damping coefficient on the predicted maximum pore depth. The results show only minor differences within the physically reasonable range considered, indicating that the main melt‐pool and keyhole dynamics are not sensitive to the exact choice of $D_{c}$.

Room‐temperature solid densities of Cu and Al were used in the inversion. To estimate the uncertainty introduced by neglecting temperature‐dependent density variations, we compared the reconstructed Cu mass fraction using two limiting density sets: room‐temperature solid densities ($\rho_{\mathrm{Cu}}$ = 8.96 g/${\mathrm{cm}}^{3}$, $\rho_{\mathrm{Al}}$ = 2.70 g/${\mathrm{cm}}^{3}$) and liquid densities at the boiling temperature ($\rho_{\mathrm{Cu}}$ = 7.90 g/${\mathrm{cm}}^{3}$, $\rho_{\mathrm{Al}}$ = 2.28 g/${\mathrm{cm}}^{3}$). This comparison provides a conservative upper‐bound estimate of the systematic uncertainty associated with neglecting temperature‐dependent density variations. Under the present experimental conditions, the maximum absolute uncertainty in the reconstructed Cu mass fraction was estimated to be below 0.02 (a 10% relative error shown in Figure 7a). Since most of the melt pool is expected to remain at temperatures well below the boiling point, the actual uncertainty is expected to be smaller than this upper‐bound estimate.

### 5.4 High‐Fidelity Process Modeling

#### 5.4.1 Multi‐Physics CFD Model

Numerical simulations were conducted for two experimental configurations with powder bed thicknesses of 50 and 150 μm. The computational domain, mesh, and coupled multi‐physics model are shown in Figure 6b. Aluminum powder particles were first generated using DEM and subsequently converted into a finite‐volume discretization for multi‐physics CFD simulations [47].

The molten material was modeled as an incompressible, laminar, Newtonian fluid. Conservation equations for mass, momentum, and energy were solved while accounting for multi‐material mixing. The momentum equation includes contributions from surface tension, Marangoni forces, recoil pressure, and buoyancy. The energy equation incorporates laser absorption, heat conduction, surface convection, thermal radiation, latent heat effects, and vaporization.

Due to the high laser power required for material mixing in multi‐material LPBF, keyhole formation is frequently observed. To accurately resolve deep melting and multiple laser reflections within the keyhole, a ray‐tracing‐based heat source model was employed, as described below [41]. Interfaces between the shielding gas and the two metallic phases were tracked using the Volume of Fluid (VoF) method [59].

Adiabatic and constant‐temperature boundary conditions were applied for thermal fields, while zero‐gradient velocity and total‐pressure boundary conditions were imposed for flow variables. Evaporation‐induced mass loss and recoil pressure effects were explicitly included. A uniform mesh size of 5 μm was used to resolve powder geometries and keyhole morphology. Thermophysical properties of all materials are provided in Table 1.

**TABLE 1:** Properties of Cu, Al, and shielding gas.

Material property	Unit	Cu	Al
Density	$\mathrm{kg}\cdot m^{-3}$	8100	2391
Solidus temperature	K	1350	928
Liquidus temperature	K	1357	933
Boiling point	K	2835	2792
Latent heat of fusion	$\mathrm{kJ}\cdot {\mathrm{kg}}^{-1}$	205	397
Latent heat of evaporation	$\mathrm{kJ}\cdot {\mathrm{kg}}^{-1}$	4720	10870
Coefficient of expansion	1$\cdot K^{-1}$	$1.7\times 10^{-5}$	$2.4\times 10^{-5}$
Viscosity	$\mathrm{kg}\cdot m^{-1}\cdot s^{-1}$	$4.2\times 10^{-5}$	$1.3\times 10^{-5}$
Molar mass	$\mathrm{kg}\cdot m{\mathrm{ol}}^{-1}$	0.0634	0.02698
Specific heat capacity (solid)	$J\cdot {\mathrm{kg}}^{-1}\cdot K^{-1}$	385	900
Specific heat capacity (liquid)	$J\cdot {\mathrm{kg}}^{-1}\cdot K^{-1}$	481	1080
Thermal conductivity (solid)	$W\cdot m^{-1}\cdot K^{-1}$	320	185
Thermal conductivity (liquid)	$W\cdot m^{-1}\cdot K^{-1}$	157	89
Surface tension	$\mathrm{kg}\cdot s^{-2}$	1.31$-1.27\times 10^{-4}$ *T*	0.874$-1.76\times 10^{-4}$ *T*
**Gas parameter**	**Unit**	**Value**	
Ambient pressure	Pa	$10^{5}$	—
Thermal conductivity	$W\cdot m^{-1}\cdot K^{-1}$	0.02	—
Viscosity	$\mathrm{kg}\cdot m^{-1}\cdot s^{-1}$	2.23 $\times 10^{-5}$	—
Density	$\mathrm{kg}\cdot m^{-3}$	1.67	—

All simulations were performed using an in‐house solver implemented in OpenFOAM and executed on an Intel Core i7‐13700K processor. The CFD formulation and multi‐material mixing model are provided as follows. The mass conservation equation is given by

(1) $$\frac{\partial \rho }{\partial t}+\nabla \cdot \left(\rho U\right)=0$$

where *t* is the time and *U* is the fluid velocity. ρ is the volume‐averaged density according to VoF. The multi‐material representation with VoF as developed by Tang et al. [41] is adopted in this work, where volume fractions of different phases (different materials and shielding gas) are denoted by the scalar variable $\alpha_{i}$, i.e., *i* = 1, 2,...*N*‐1 for metals and *i* = *N* for the gas. Consequently, the VoF equations for metallic materials and gas are written as follows, respectively.

(2) $$\frac{\partial \alpha_{i}}{\partial t}+\nabla \cdot \left(U\alpha_{i}\right)+\nabla \cdot \left(\alpha_{i}\alpha_{N}U_{r}\right)=\nabla \cdot \left[\left(D_{l}\gamma \alpha_{m}\right)\nabla \frac{\alpha_{i}}{\alpha_{m}}\right]$$

(3) $$\frac{\partial \alpha_{N}}{\partial t}+\nabla \cdot \left(U\alpha_{N}\right)-\nabla \cdot \left[\alpha_{N}\left(1-\alpha_{N}\right)U_{r}\right]=0$$

$U_{r}$ represents the artificial relative velocity, $\alpha_{m}$ = $\sum_{i=1}^{N}\alpha_{i}$ is the total volume fraction of metallic materials, and γ is a temperature dependent function according to the solidus and liquidus points of the materials. $D_{l}$ denotes the effective liquid solute diffusivity in the bulk liquid. Experimental studies indicate that diffusivities in liquid metals near the melting point are typically of the order of $10^{-9}\;m^{2}/s$ [60]. Consistent with this order of magnitude, Zaeem et al. [61] adopted $D_{l}$ = $3\times 10^{-9}\;m^{2}/s$ for an Al‐Cu system, which is similar to the system considered in the present work. Since material mixing under the present LPBF conditions is governed primarily by melt‐pool convection and keyhole‐induced flow, whereas molecular diffusion plays a secondary role, a constant value of $D_{l}$ = $3\times 10^{-9}\;m^{2}/s$ was adopted here as a pragmatic first‐order approximation for the bulk‐liquid solute transport term.

The momentum conservation equation for the thermal fluid flows is given by

(4) $$\begin{array}{cc} & \frac{\delta }{\delta t}\left(\rho U\right)+\nabla \cdot \left(\rho U\times U\right)\\ & \;=\nabla \cdot \left(\mu \nabla U\right)-\nabla p_{rgh}-\rho g+F_{s}+F_{m}+P_{r}+F_{damp} \end{array}$$

where μ is the dynamic viscosity averaged in volume, $p_{rgh}$ is the dynamic pressure, *g* is the gravitational acceleration. $F_{s}$, $F_{m}$, $P_{r}$, and $F_{damp}$ represent the summation of surface tension forces, summation of recoil pressure, summation of Marangoni forces, and the damping term, respectively. These source terms in the multi‐material framework are presented in the following section.

With consideration of the laser heat source, latent heat of vaporization, viscous dissipation, thermal convection, latent heat of melting, and radiation, the energy conservation equation is given by

(5) $$\begin{array}{cc} & \frac{\partial }{\partial t}\left(C_{p}\rho T\right)+\nabla \left(C_{p}\rho UT\right)-\nabla \cdot \nabla \left(kT\right)\\ & \;=-L_{m}\left[\frac{\partial \rho \gamma }{\partial t}+\nabla \cdot \left(\rho U\gamma \right)\right]+Q_{laser}+Q_{rad}+Q_{vap} \end{array}$$

where $L_{m}$ represents the latent heat of fusion. The volume averaged thermal conductivity and specific heat capacity are written as follows, respectively.

(6) $$k=\sum_{i=1}^{N}\alpha_{i}\left[\left(1-\gamma \right)k_{s,i}+\gamma k_{l,i}\right]$$

(7) $$\rho C_{p}=\sum_{i=1}^{N}\alpha_{i}\rho_{i}C_{p,i}$$

$k_{s,i}$ and $k_{l,i}$ are the thermal conductivities of the solid and liquid phases of the material *i*. $C_{p,i}$ is the capacity of material *i*. Other heat sources and losses will be explained in the following section.

To further assess the robustness of the VoF treatment, we quantified both mesh sensitivity and phase conservation. As shown in Figure 7b, the temporal evolution of the total volume fraction was evaluated for three mesh sizes, Δx = 7.5, 5.0, and 2.5 μm. In all cases, the summed volume fraction remains essentially equal to the theoretical value, and the deviation decreases with mesh refinement. For the finest mesh, the error is on the order of $10^{-6}$, indicating excellent inter‐phase mass conservation.

Figure 7c further shows the temporal evolution of the globally integrated gas, Cu, and Al volume fractions. These phase fractions remain nearly constant throughout the simulation, showing that numerical diffusion across the interfaces is very limited in the present multiphase formulation. Therefore, the predicted composition field and pore evolution are not significantly affected by artificial interface diffusion.

#### 5.4.2 Source Terms in the Multiphysics Model

In the momentum conservation equation, the external forces are expressed in summation form, as introduced in Ref. [41]. As the materials are miscible, there is no need to account for the surface tension and Marangoni forces between two metallic materials, while the interfacial forces between metals and gas should be calculated. The summation of surface tension is given by

(8) $$F_{s}=\sum_{i=1}^{N-1}2\alpha_{m}\varepsilon_{i}\kappa_{i}\left(\alpha_{N}\nabla \alpha_{i}-\alpha_{i}\nabla \alpha_{N}\right)$$

where $\varepsilon_{i}$ represents the surface tension coefficient between the material *i* and the gas phase. $\kappa_{i}$ is the interface curvature between the material *i* and the gas, which is written as ‐$\nabla \cdot n_{i}$ with $n_{i}$ denoting the interface normal vector.

The summation of Marangoni force is written as

(9) $$F_{m}=\sum_{i=1}^{N-1}2\alpha_{m}\frac{d\varepsilon_{i}}{dT}\left[\nabla T-n_{i}\left(n_{i}\cdot \nabla T\right)\right]\left(\alpha_{N}\nabla \alpha_{i}-\alpha_{i}\nabla \alpha_{N}\right)$$

where $\frac{d\varepsilon_{i}}{dT}$ represents the surface tension gradient.

Similarly, the summation of recoil pressure is obtained as

(10) $$P_{r}=\sum_{i=1}^{N-1}2\Gamma \alpha_{m}p_{0}\exp\left(\frac{l_{v,i}M_{i}\left(\frac{1}{T_{v,i}}-\frac{1}{T}\right)}{R}\right)\left(\alpha_{N}\nabla \alpha_{i}-\alpha_{i}\nabla \alpha_{N}\right)$$

where Γ shows a fraction of the saturated vapor pressure and is adopted as 0.54 in this work (see our work [47] for details on choosing this value). $p_{0}$ is the initial pressure. *R* is the gas constant. $l_{v,i}$, $M_{i}$, and $T_{v,i}$ represent the latent heat of vaporization, molar mass, and vaporization temperature of metal *i*.

The Darcy damping term is shown as

(11) $$F_{damp}=\rho D_{c}\frac{{\left(1-\gamma \right)}^{2}}{\gamma^{3}+D_{cs}}U$$

where $D_{cs}$ is adopted as a small constant value to avoid singularities. It is typically set to $10^{-5}$, and its influence on the simulation results is negligible. By contrast, $D_{c}$ is a large empirical coefficient related to the permeability of the molten metal in the mushy region, and is generally taken in the range of $10^{5}$–$10^{6}$. Within this range, the predicted pore depth remains stable, indicating that the main melt‐pool dynamics are not sensitive to the exact value of $D_{c}$ as long as it remains within a physically reasonable interval, as shown in Figure 7d. Similar tests have been reported in Ref. [62], where $D_{c}$ varies over a wider range (from 10 to $10^{9}$), yet the simulation results show little difference.

Moreover, the heat sources and losses considered in the energy conservation equation are shown as follows. We adopted a ray tracing model to calculate the laser heat source $Q_{laser}$, which is very important to accurately simulate keyhole evolution. The summation of radiation heat losses of different materials is given by

(12) $$Q_{rad}=\sum_{i=1}^{N-1}-2\alpha_{m}\sigma \eta_{i}\left(T^{4}-T_{ref}^{4}\right)\left|\alpha_{N}\nabla \alpha_{i}-\alpha_{i}\nabla \alpha_{N}\right|$$

where σ denotes the Stefan–Boltzmann constant, $\eta_{i}$ is the radiation emissivity, and the ambient temperature $T_{ref}$ is 300 K. Finally, the heat loss caused by evaporation is given by

(13) $$\begin{array}{cc} & Q_{vap}\\ & =\sum_{i=1}^{N-1}-1.21\Gamma \alpha_{m}\frac{l_{v,i}M_{i}}{\sqrt{M_{i}RT}}p_{0}\exp\left(\frac{l_{v,i}M_{i}\left(\frac{1}{T_{v,i}}-\frac{1}{T}\right)}{R}\right)\left(\alpha_{N}\nabla \alpha_{i}-\alpha_{i}\nabla \alpha_{N}\right) \end{array}$$

#### 5.4.3 Multi‐Material Ray Tracing Model

Laser–material interaction was modeled here using a ray‐tracing approach that accounts for real‐time Fresnel reflections at material interfaces [63]. The model consists of two steps: initialization of the laser beam and discretization into individual rays, followed by tracing laser rays to determine absorption and reflection.

A Gaussian laser intensity distribution was assumed, calculating

(14) $$I_{L}=\frac{\zeta P_{L}}{\pi r^{2}}\exp\left[-\zeta \frac{{\left(X-U_{0}t-X_{0}\right)}^{2}+{\left(Y-Y_{0}\right)}^{2}}{r^{2}}\right]$$

where $P_{L}$ is laser power, *r* the beam radius, ζ the Gaussian distribution factor set to 3 in this work, $U_{0}$ the scanning speed, (*X*, *Y*) the current beam position, and ($X_{0}$, $Y_{0}$) the initial beam position. Consequently, each laser ray was initialized with four variables: position **X**, propagation direction $n_{i}$, power $P_{L,i}$, and frequency of reflection $f_{r}$. The power of laser rays located in a finite domain around the laser spot point was assigned as

(15) $$P_{L,i}=\frac{3P_{L}\Delta x^{2}}{\pi r^{2}}\exp\left[-3\frac{{\left(X_{i}-U_{0}t-X_{0}\right)}^{2}+{\left(Y_{i}-Y_{0}\right)}^{2}}{r^{2}}\right]$$

where Δx denotes the numerical grid spacing.

To track the trajectory of a laser ray, the reflectivity ξ at material interfaces was computed as a function of the incident angle θ using Fresnel equations

(16) $$\xi =\frac{1}{2}\left[\frac{{\left(n_{i}\cos\theta -1\right)}^{2}+k_{i}^{2}\cos^{2}\theta }{{\left(n_{i}\cos\theta +1\right)}^{2}+k_{i}^{2}\cos^{2}\theta }+\frac{{\left(n_{i}-\cos\theta \right)}^{2}+k_{i}^{2}}{{\left(n_{i}+\cos\theta \right)}^{2}+k_{i}^{2}}\right]$$

where $n_{i}$ and $k_{i}$ are the real and imaginary parts of the refractive index of the material interacting with ray *i*. To avoid confusion, the 19 keV value reported above refers only to the synchrotron X‐ray beam energy used for operando radiography. By contrast, the ray‐tracing model describes laser energy deposition and is governed by the laser wavelength. In the present model, laser absorption is evaluated through the Fresnel equations using the complex refractive index (*n*, *k*) of the material at the laser wavelength [64]. These two optical constants are treated as temperature independent in most ray‐tracing models, because introducing temperature‐dependent optical properties would require update for each ray–surface interaction, which would substantially increase the computational cost. Nevertheless, the ray‐tracing formulation still provides a more realistic description of laser energy deposition than a conventional volumetric Gaussian heat source, especially under keyhole‐dominated conditions [38, 65].

The local laser heat source term in the energy equation was then updated as

(17) $$Q_{laser}\left(i,j,k\right)=Q_{laser}^{old}\left(i,j,k\right)+\left(1-\xi \right)\frac{P_{L,i}}{\Delta x^{3}}$$

Details of ray–surface intersection detection and its numerical implementation within a finite‐volume framework are not repeated here and can be found in previously published works, e.g., Tang et al. [41].

## Author Contributions

**Zhilang Zhang**: conceptualization, investigation, methodology, software, visualization, supervision, writing – original draft. **Steve Gaudez**: experimental campaign, sample preparation and analysis, post‐processing, writing – original draft. **Mario Togni**: investigation, software, visualization. **Steven Van Petegem**: experimental campaign, contribution to data interpretation, writing – review & editing. **Markus Bambach**: supervision, writing – review & editing, project administration, funding acquisition. **Mamzi Afrasiabi**: conceptualization, investigation, methodology, visualization, supervision, writing – original draft, writing – review & editing, project administration.

## Conflicts of Interest

The authors declare no conflicts of interest.

## Supporting information


**Supporting File 1**: advs75723‐sup‐0001‐VideoS1.avi.

**Supporting File 2**: advs75723‐sup‐0002‐VideoS2.avi.

## Data Availability

The data that support the findings of this study are available from the corresponding author upon reasonable request.
